# The safety and quality of childbirth in the context of health systems: mapping maternal health provision in Lebanon

**DOI:** 10.1016/j.midw.2010.06.012

**Published:** 2010-10

**Authors:** Jocelyn DeJong, Chaza Akik, Faysal El Kak, Hibah Osman, Fadi El-Jardali

**Affiliations:** a Department of Epidemiology and Population Health, Faculty of Health Sciences, American University of Beirut, Beirut, Lebanon; b Faculty of Health Sciences, American University of Beirut, Beirut, Lebanon; c Department of Health Behavior and Education, Faculty of Health Sciences, American University of Beirut, Beirut, Lebanon; d Department of Health Management and Policy, Faculty of Health Sciences, American University of Beirut, P.O. Box 11-0236, Riad El Solh, 1107 2020 Beirut, Lebanon

**Keywords:** Maternal health, Safety, Health system

## Abstract

**Objective:**

to provide basic information on the distribution (public/private and geographically) and the nature of maternity health provision in Lebanon, including relevant health outcome data at the hospital level in order to compare key features of provision with maternal/neonatal health outcomes.

**Design:**

a self-completion questionnaire was sent to private hospitals by the Syndicate of Private Hospitals in collaboration with the study team and to all public hospitals in Lebanon with a functioning maternity ward by the study team in cooperation with the Ministry of Public Health.

**Setting:**

childbirth in an institutional setting by a trained attendant is almost universal in Lebanon and the predominant model of care is obstetrician-led rather than midwife-led. Yet due to a 15-year-old civil war and a highly privatised health sector, Lebanon lacks systematic or publically available data on the organisation, distribution and quality of maternal health services. An accreditation system for private hospitals was recently initiated to regulate the quality of hospital care in Lebanon.

**Participants:**

in total, 58 (out of 125 eligible) hospitals responded to the survey (46% total response rate). Only hospital-level aggregate data were collected.

**Measurements:**

the survey addressed the volume of services, mode of payment for deliveries, number of health providers, number of labour and childbirth units, availability of neonatal intensive care units, fetal monitors and infusion rate regulation pumps for oxytocin, as well as health outcome data related to childbirth care and stillbirths for the year 2008.

**Findings:**

the study provides the first data on maternal health provision from a survey of all eligible hospitals in Lebanon. More than three-quarters of deliveries occur in private hospitals, but the Ministry of Public Health is the single most important source of payment for childbirth. The reported hospital caesarean section rate is high at 40.8%. Essential equipment for safe maternal and newborn health care is widely available in Lebanon, but over half of the hospitals that responded lack a neonatal intensive care unit. The ratio of reported numbers of midwives to deliveries is three times that of obstetricians to deliveries.

**Key conclusions and implications for practice:**

there is a need for greater interaction between maternal/neonatal health, health system specialists and policy makers on how the health system can support both the adoption of evidence-based interventions and, ultimately, better maternal and perinatal health outcomes.

## Introduction

Global efforts to reduce maternal mortality – including the millennium development goals – have focused on increasing trained attendance at childbirth (Campbell and Graham, 2006). Such a strategy would also reduce neonatal deaths (Darmstadt et al., 2005). This approach indirectly encourages institutional childbirth but assumes that facility-based quality of care avoids harm and is life-saving. The actual quality of care – for both normal deliveries, which constitute the majority of deliveries worldwide, and those with complications – has received relatively less research attention than other areas of health care (Althabe et al., 2008). Quality of maternal health internationally has been described as a ‘neglected’ agenda in maternal health (Van den Broek and Graham, 2009). Moreover, maternal and neonatal care has been identified as one of the top 20 patient safety problems in developing countries and for economies in transition (World Health Organization Patient Safety, 2008).

Recommendations to improve practice in order to enhance patient safety and quality need to be grounded in knowledge of available structures and processes in health-care delivery in order to be able to link these to actual health outcomes. It is increasingly recognised that promoting safety and quality in maternal health is dependent on the overall functioning of health systems (Penn-Kekana et al., 2007; Bhutto et al., 2008), but the contribution of health system factors to maternal health has not been well specified to date (Parkhurst et al., 2005) This is particularly the case for middle-income countries, given that most of the literature on health system impacts on maternal health focuses on settings where human resource constraints are severe and access to obstetric care is poor, such as in low-income countries affected by human resource losses due to human immunodeficiency virus/acquired immunodeficiency syndrome or to outward migration to richer countries (e.g. Gerein et al., 2006; Turan et al., 2008). There are, however, evident problems of quality in many countries with relatively good coverage of services, both in terms of patient safety/health outcomes and women’s experiences of childbirth (Miller et al., 2003; Hulton et al., 2007). High coverage is not sufficient without good quality of care to improve maternal/newborn health outcomes (Countdown Working Group on Health Policy and Health Systems, 2008).

In countries of the Middle East, access to a health professional for childbirth care is no longer a key constraint; the large majority of women now deliver with doctors or midwives in institutional settings (World Health Organization Department of Reproductive Health and Research, 2008). The region, in general, has high access to hospital obstetric care mainly by clinicians, but with gender imbalances and a deficiency in the number of other categories of providers, midwives and nurses, who might contribute to an effective, sustainable and satisfying model of care for normal childbirth (Hatem et al., 2008). The multidisciplinary regional research network, the Choices and Challenges in Changing Childbirth Research Network^1^ established in 2001, has documented problems in quality and safety across five countries of the Middle East region including Lebanon (Choices and Challenges in Changing Childbirth Research Network, 2005; Sweidan et al., 2008). Studies have found that the reasons for these safety and quality deficiencies, however, are complex and highly dependent on different health system factors that warrant further exploration.

In the specific case of Lebanon, the country is well endowed with facilities and medical manpower, and access to childbirth care is high (see Table 1). However, a study of a nationally representative sample of hospitals in the country documents the lack of adherence to evidence-based recommendations in a number of areas of maternal health provision, with some widespread practices being potentially harmful (Khayat and Campbell, 2000). Practising clinicians from the Network further report that augmentation and induction of labour with oxytocin is a widespread practice.^2^ Analysis of population-based data and hospital-based studies by the Network has also revealed an excessive rate of caesarean section at the population level in Lebanon, well above the World Health Organization recommended maximum of 15% (Jurdi and Khawaja, 2004; Khawaja et al., 2004). Moreover, the most recent nationally representative population-based survey for Lebanon, fielded in 2004, indicates that maternal mortality is relatively high given the middle-income level of the country and the high availability of health care (Tutelian et al., 2007), although studies currently underway suggest that it is likely to be much lower. Finally, qualitative research by network members with women who had recently delivered in Lebanon revealed that many encounters with the maternity services are negative and that some routine hospital practices are uncomfortable for women (Kabakian-Khasholian et al., 2000).

At the same time, however, Lebanon lacks basic information about the organisation, distribution and quality of maternal health services, and hospital-level maternal and newborn health outcome data are neither publically available nor systematically collected.^3^ These deficiencies in the information base are due to the inter-related factors of the legacy of a 15-year civil war (1975–1990) and the limited role of the state in a health sector heavily dominated by the private sector. This paper reports on findings from the first part of a three-pronged study investigating health system factors affecting the quality and safety of maternal health delivery in Lebanon. This first stage consists of a mapping of available hospital-based childbirth services in the public and private sectors given the absence of available information on maternal health-care provision and maternal/neonatal health outcomes in the country. The study aims to provide basic national information on the distribution (public/private and geographically) and the nature of maternity health provision (including both human and physical resources) and relevant health outcome data at the hospital level in order to compare key features of provision and maternal/neonatal health outcomes. The other study components (currently underway) consist of a survey of heads of maternity wards concerning hospital practices and policies to update and expand upon earlier research (Khayat and Campbell, 2000), and qualitative research with providers (both clinicians and nurses/midwives). This paper will first present the health system context of Lebanon and the organisation of maternity care before turning to the study methods, findings and discussion of the implications of the research findings for policy, practice and research.

## The health system/health policy context in Lebanon

Lebanon has a large pluralistic and highly fragmented health sector where several players are involved in the financing and provision of health-care services to the population (Mohammad Ali Osseiran et al., 2005). The private sector flourished as a result of the Civil War (1975–1990) which saw many of the existing public sector hospitals destroyed, leading to the marginalisation of the public sector and, as such, the role of the Government in the delivery of health services. It should be noted, however, that private hospitals are highly dependent on revenue from public funding, mainly the Ministry of Public Health (MOPH) in addition to other ministries including the Ministry of Labour, the Ministry of Defence, the Ministry of Interior and Office of the Prime Minister (Mohammad Ali Osseiran et al., 2005; Ammar, 2009). The proliferation of the private sector led to several challenges including: weakening of the MOPH, inflation of the medical bill due to emphasis on curative care and high-technology medical care, weakening of primary care, and oversupply of health facilities and their concentration in urban areas (Mohammad Ali Osseiran et al., 2005).

In one of the only comprehensive recent studies of the health system in Lebanon, Ammar (2009) notes that the overwhelming majority of the estimated 168 hospitals in Lebanon is privately owned. He further reports that in spite of the recent establishment of 28 public hospitals, private hospitals represent around 80% of all hospitals in Lebanon, and the prices of public hospitals are 10% lower than those of private hospitals. Public hospitals do not operate to generate profit, but rather aim to break even which creates incentives to attract patients and reduce transfer to private hospitals. As such, admissions to public hospitals increased between 2005 and 2007 (Ammar, 2009).

Despite many attempts at gaining some form of control, the MOPH has limited authority and regulation over care provision in private hospitals (Mohammad Ali Osseiran et al., 2005; Ammar, 2009). Private hospitals enjoy the luxury of being able to invest in areas that allow them to maximise profit. As such, they are less concentrated in rural areas as urban areas are more profitable. This has led to severe regional discrepancies which are reflected in inequitable service provision across geographic regions. The distribution of public hospitals is believed to be more equitable than private hospitals, although they only comprise 2550 beds (Ammar, 2009). In 1999, the Lebanese Syndicate of Private Hospitals reported an estimated 9297 active beds in private hospitals in Lebanon (Mohammad Ali Osseiran et al., 2005). Recent estimates indicate that the number of hospital beds is 2.9 beds per 1000 population, which is believed to be comparable to rates in developed countries and exceeds the rate in other countries in the region (Mohammad Ali Osseiran et al., 2005). The Lebanese Syndicate of Private Hospitals, on the other hand, estimates the bed per population ratio at one bed for every 255 people (Mohammad Ali Osseiran et al., 2005).

### Accreditation

In an effort to improve the quality of hospital care and establish regulation mechanisms, the hospital accreditation policy was enacted in 2002. With the assistance of an Australian consultant team, the MOPH developed and implemented a process of evaluating the quality of care in terms of processes of care, rather than outcomes. This new policy, which replaced the old classification system which had been in use since 1983, was implemented in four phases (Ammar et al., 2007). The MOPH is now using this policy as an incentive-based regulation by implementing a payment system which links accreditation to reimbursement (Ammar, 2009).

The MOPH implemented accreditation of private hospitals through two national surveys, the first between September 2001 and July 2002 and the second in 2004–2005 (Ammar et al., 2007; Ammar, 2009). The second survey which included 142 hospitals found that only 85 hospitals met requirements. A study aimed at assessing the impact of accreditation on quality of care as perceived by nurses showed that nurses believed it to be a good tool for improving quality of care, and perceived quality of care was found to have improved (El-Jardali et al., 2008).

### Human resources

The health-care sector is both labour-intensive and labour-reliant, and the delivery of quality health-care services is strongly dependent on having enough well-trained health-care workers to meet patient needs and expectations (El-Jardali et al., 2007). As with most countries, Lebanon is suffering from critical problems with its health workforce which can affect health-care delivery. The most pressing problem is the imbalance between clinicians and nurses. Recent evidence indicates that the ratio of clinicians is almost three times that of nurses in Lebanon (El-Jardali et al., 2007). The country is also believed to be suffering from a shortage of nurses including midwives (Ammar, 2009). This is critical as recent evidence links the health provider density with population outcomes. In fact, increasing clinician density was found to be linked to lower maternal mortality, infant mortality and under-5 mortality. Improving nurse density was linked to lower maternal mortality rates (El-Jardali et al., 2007). However, it should be noted that such improvements are more pronounced in countries of higher income classification, which might imply that there are other critical predictors that are as important as the overall number of health-care providers that can improve population outcomes in countries of lower income classification (El-Jardali et al., 2007).

## Maternal health care in Lebanon

According to the most recent nationally representative population-based survey at the household level that included questions on maternal health (Tutelian et al., 2007), childbirth with a skilled attendant is almost universal in Lebanon: 98.2% of births in the preceding five years (see Table 1). Most births take place in hospital, with private hospitals accounting for 80.1% of all deliveries within the five years preceding the survey. Antenatal care is relatively high (with 70.5% of respondents having made five or more antenatal visits), but only roughly half of the respondents had any postnatal care. The population-based caesarean section rate was found to be high at 23.2% (Table 1).

Maternal health in Lebanon, like other areas of health care, has historically been, and remains, dominated by the medical profession. This can be traced to the highly privatised nature of the health system and the fact that private clinicians are the main agents of medical care. This is particularly the case in maternal health, which is viewed, in the medicalised culture of the country, as an important and private matter that should be entrusted to clinicians’ professional skills (obstetricians). There has been an unregulated increase in the number of medical doctors (from both genders) seeking residency programmes and training in obstetrics and gynaecology. Today, according to the Order of Physicians in Lebanon, the estimated number of registered obstetricians is close to 950, with about 650–700 currently practising in Lebanon. This dominant pattern of maternal health care being obstetrician-led has consistently marginalised midwifery, which has been losing the narrow grounds occupied historically in private practice (in the 1960–1980s). Midwives are now mainly relegated to the role of assisting obstetricians in labour and childbirth within hospitals. Lebanese law stipulates that maternity patients cannot register in hospitals under the name of midwives, but must do so under the name of obstetricians. Pregnant women tend to stay with the provider who deals with conception, pregnancy care, ultrasound examinations and related procedures, and all the other aspects of medicalised care. This situation has reduced the chances of midwives being alternative providers of maternal health care with competitive benefits, especially that private obstetric providers have opportunities to work in accessible, subsidised health centres and dispensaries, either governmental or non-governmental, eliminating the financial obstacles to care that historically favoured midwives.

The differential status of the midwifery profession has further contributed to this imbalance. The Lebanese Society of Obstetrics and Gynaecologists actively promotes that profession in terms of administrative, organisational and scientific issues. In contrast, midwives – due to political and professional reasons – have not yet succeeded in organising and registering a professional society that could promote their profession and the right to lead/co-lead maternal health care. There is increased competition over the provision of maternal health care given the privatised nature of the health system, the declining birth rate in the country (with the total fertility rate below replacement level) and the market-driven increase in the number of obstetricians in the absence of any regulation on specialties – which have together exacerbated these differentials between the two professions.

Nevertheless, the recently introduced process of accreditation of private hospitals could potentially contribute to an enhanced status for midwives in the country. The latest accreditation criteria for maternity wards stipulate that obstetric departments should be under the direction of a qualified obstetrician, but be managed by a ‘registered midwife qualified by education and a minimum of five years experience’ (Criterion OB2, Ministry of Public Health, 2003), and that ‘the staff schedule provides for at least one registered general nurse with midwifery qualifications on duty’ (Criterion OB3.4, Ministry of Public Health, 2003).

## Methods

This study was conducted in collaboration with the MOPH and the Syndicate of Private Hospitals which have real or *de facto* jurisdiction, respectively, over the public and private hospitals in Lebanon. In February 2009, the Syndicate of Private Hospitals sent out, via fax, questionnaires to all private hospitals in Lebanon that are members of the Syndicate (*n*=127 of which *n*=108 proved to be eligible with a maternity ward), accompanied by an explanatory letter, and requested that they be completed and returned to the Syndicate within two weeks of receipt. For public hospitals, all operational hospitals in the country were contacted in order to identify their eligibility in terms of having a functioning maternity ward. In June 2009, questionnaires were sent out by fax to all eligible public hospitals by the study team with an explanatory letter signed by the MOPH which endorsed the study; hospitals were asked to return the completed questionnaires to the study team within two weeks of receipt. The administration of all study hospitals were asked to assign a focal person who would collect the information from relevant departments. In total, the questionnaire was sent to 144 hospitals. Given the voluntary nature of the survey and the anticipated reluctance of many hospitals – particularly private hospitals – to reveal hospital-based statistics, and in order to increase the response rate, follow-up phone calls were conducted in April and mid-June 2009 for private and public hospitals, respectively, with administrators to confirm receipt of the questionnaire and to encourage their completion and return.

### Ethical considerations

This study was submitted for ethical review by the Institutional Review Board at the American University of Beirut, and granted exemption as it involved the collection of aggregate statistics available at hospitals.

### Questionnaire design

The survey instrument was developed and refined by a multidisciplinary study team combining expertise in health policy, maternal health, the Lebanese health system and clinical experience in family medicine and obstetrics in Lebanese hospitals. The questionnaire was developed in English then translated into Arabic. The Arabic version was reviewed by both the Syndicate of Private Hospitals and the MOPH. The main components of the survey address the volume of services, mode of payment for deliveries, number of health providers, number of labour and childbirth units, and the volume of the different types of deliveries and availability of infusion rate regulation pumps (used to regulate the administration of oxytocin) and neonatal intensive care units (NICU) for 2008. Health outcome data at the hospital level were requested on caesarean section (including for primigravidae and multigravidae), instrumental deliveries, numbers of stillbirths and live births. Given the reluctance of hospitals generally in Lebanon to disclose health outcome data, particularly in a context of an on-going accreditation process for private hospitals, which is process rather than outcome orientated, it was deemed too sensitive to collect data on maternal and neonatal mortality that could have jeopardised the response rate. Given the highly privatised and competitive environment of the health system in Lebanon, hospitals do not readily release patient outcome data for research purposes and are not required to make this data available in the public domain.

### Data analysis

Data were entered and analysed using Statistical Package for the Social Sciences Version 16.0 for Windows. Univariate analysis was performed to obtain a general description of the volume of services, payment coverage, number of health providers, number of labour and childbirth units, and the volume of the different types of deliveries in hospitals. *t*-Tests were performed to assess the relationship between the indicators stated above and hospital type and location. *p*-Values were computed for all above tests with *p*≤0.05 considered to be statistically significant.

## Findings

Out of 108 eligible private hospitals in Lebanon, 46 responded and thus the private hospital response rate was 43.0%.^4^ Out of the 17 eligible public hospitals in the country, 12 responded giving a response rate for the public hospitals of 70.6%. In total, the 58^5^ (out of 125 eligible) hospitals which responded to the survey (46% total response rate) provided data on 35,883 deliveries which occurred in their settings in 2008 (see Table 2 on the numbers of deliveries, live births and stillbirths by type and region of hospitals that responded to the survey). Table 3 presents the role within the study hospitals of the respondents to the surveys; some hold multiple roles within hospitals, depending on hospital size and available human resources, but all those who responded to the survey are knowledgeable about maternal health.

The private sector accounted for the majority of deliveries in 2008 in the respondent hospitals: 77.8% of deliveries vs 22.2% in the public sector (Table 4). Table 5 shows the distribution of payments for deliveries by different modes of payment, allowing for the fact that deliveries could be paid for by multiple sources and therefore the denominator does not correspond to the number of deliveries. Payment by the MOPH is the biggest single source of payment for childbirth, accounting for 36.8% of all modes of payment for childbirth, but the MOPH accounts for 85.3% of modes of payment for childbirth within public hospitals and 21.9% within private hospitals (Table 5).

The mean reported caesarean section rate was found to be 40.8% across all hospitals, with a negligible (and non-significant) difference in the reported rate between the public (40.2%) and private sectors (41.0%) and between hospitals within the capital city, Beirut, and its suburbs (38.7%) compared with reporting hospitals in the rest of the country (41.7%) (see Table 6).

Vaginal instrumental deliveries account for 10.7% of all reported deliveries overall, with the reported percentage being significantly higher within private hospitals (at 12.4%) compared with public hospitals (at 3.9%), and the same rate was higher in hospitals within Beirut and its close suburbs (at 18.0%) compared with hospitals in the rest of the country (mean rate 6.9%) (Table 7).

Comparisons of means revealed significant differences in the caesarean section rate between private hospitals accredited in 2004 (mean of 44.4%) compared with non-accredited hospitals (mean of 31.4%) (Table 8). However, although there was a difference in the reported use of instrumental deliveries between those that passed accreditation (at 13.1%) and those that did not (at 7.0%), this difference was not statistically significant. Taking instrumental deliveries alone, accredited hospitals reported a significantly higher rate of use of forceps (at 41.0%) than the non-accredited hospitals (at 8.3%). An inverse relationship applies for the use of vacuum extraction, whereby accredited hospitals reported a rate of 56.7% among instrumental deliveries, as opposed to 91.7% for non-accredited hospitals.

The mean ratio of the annual number of deliveries to the numbers of midwives with delivery privileges employed in each hospital is about three times higher than the same ratio for obstetricians (Table 9), with no significant difference between public and private hospitals or between Beirut and outside Beirut (data not shown).

**Fig. 1:**
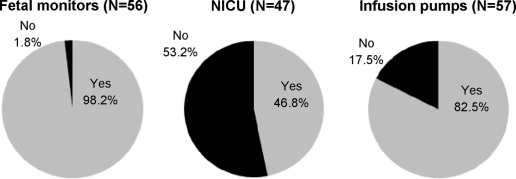
Availability of fetal monitors, neonatal intensive care units (NICU) and infusion pumps in the sampled hospitals.

Overall, 10 out of 57 hospitals (or 17.5%) reported that they did not have infusion pumps, and one hospital reported that it did not have fetal monitors (see Fig. 1). The mean availability of fetal monitors per childbirth per day (calculated as a ratio of total annual deliveries divided by 365) was found to be 3.8. The availability of infusion pumps per childbirth per day was found to be 2.7. The availability of NICUs was less, at only 0.3 per patient per day overall, but 25 out of 47 hospitals (or 53.2%) reported that they did not have an NICU. There was no significant difference in the availability of fetal monitoring, infusion pumps or NICU per childbirth per day across public versus private hospitals, or between hospitals in Beirut/close suburbs and the rest of the country (Table 10). The mean number of deliveries per labour bed per day was the same, at 0.6 in total, in the public and private hospitals and in hospitals within Beirut/its suburbs and the rest of the country (Table 10). However, the non-accredited hospitals reported a significantly lower number of deliveries per labour bed per day and a significantly lower number of deliveries per childbirth room per day than accredited hospitals (Table 8).

Hospitals that did not pass the accreditation process had a higher mean availability of fetal monitors and NICUs, and a lower mean availability of infusion pumps than hospitals that did pass, although these differences were not statistically significant (Table 8).

## Discussion

In many developing countries, particularly middle-income countries, constraints related to access to care are lessening with the increased trend towards institutional deliveries (World Health Organization Department of Reproductive Health and Research, 2008). Thus attention is needed to ensure the quality and safety of care received, and in reducing differentials in provision and quality across geographic region, public and private sectors. This appears to be the case in Lebanon where institutional deliveries are nearly universal but problems of quality have been documented; more and better information is needed on how provision relates to health outcomes and how inequities at health system level affect quality. This study makes a first step in rectifying this gap. Lebanon may be exceptional in the degree to which information about maternal health-care provision is lacking, but the problems identified in this research are likely to resonate with other developing countries where the private sector dominates and which lack a comprehensive picture of maternal health-care provision and how health system factors affect it.

The overall response rate to the survey (46% of eligible hospitals) and the deliveries reported by these hospitals account for about 42% of all live births that are reported to have taken place nationally in Lebanon in 2008 . This level of response is on a par with similar surveys of hospitals in Lebanon (El-Jardali et al., 2008, 2009a), and indicates that even in a highly fragmented, competitive and privatised health system context, it is possible to obtain data on hospital provision from a high proportion of hospitals nationally. The higher response rate for public than private hospitals illustrates the challenge of obtaining information on provision and health outcomes from the private sector particularly, despite the fact that the survey was administered by the Syndicate of Private Hospitals.

The findings from hospital-level data confirm earlier nationally representative data at a household level (Tutelian et al., 2007) that the vast majority of deliveries (77.8%) in Lebanon occur in private hospitals, and that despite efforts to increase public sector provision (Ammar, 2009), the proportion of all deliveries that take place in the public sector appears to have increased negligibly since 2004, with the proportion in this study only 2.5% higher than the national figure in 2004 (Table 1). As is the case with health care generally in Lebanon, within the public sector, the predominant mode of payment is public, and within the private sector, the predominant mode of payment is private. For this reason, the analysis by public and private sector was conducted, although it is acknowledged that the picture is complicated in that within both the public and private hospitals, deliveries are paid for from both private and public sources. The data indicate, however, that while outside Beirut, the main source of payment for deliveries was the MOPH, within Beirut and its suburbs, the main sources of payment are private. The mode of payment for childbirth is important in considering hospital administrators’ motivation for quality improvement (El-Jardali et al., 2009b).

This study found an alarmingly high mean hospital caesarean section rate, at 40.8% of all reported deliveries in 2008. A high and rising caesarean section rate is consistent with the pattern found in the region (Jurdi and Khawaja, 2004; Khawaja et al., 2004)^6^ but is much higher than the population-based rate for Lebanon of 23% in 2004 (Tutelian et al., 2007). It is also higher than other available data on hospital caesarean section rates, which found a rate of 31.4% for the period 2001–2002 in nine hospitals in Beirut participating in a neonatal database network (Tamim et al., 2007). This high hospital-based caesarean section rate is, however, consistent with more recent hospital-based studies (Osman et al., unpublished). Research by network members has found a range of factors that encourage high caesarean section in Lebanon, including lack of regulation, financial and convenience factors for clinicians, and a trend towards encouraging and accommodating women’s demand for caesarean section (Kabakian-Khasholian et al., 2007). The reasons for the high observed caesarean section rates need further exploration within individual hospitals. The topic is of particular interest to policy makers in Lebanon concerned, with encouragement by the World Bank, to rationalise health services (Shadi Saleh, American University of Beirut, personal communication, 20/11/2009). The finding that caesarean section rates are higher in accredited hospitals than non-accredited hospitals is puzzling, especially given that one of the criteria for accreditation is the availability of policies and procedures, and caesarean criteria are specifically mentioned (Criterion OB12.10, Ministry of Public Health, 2003). It may be due to the fact that accredited hospitals are likely to be the larger hospitals which also serve as referral centres. This would need to be explored in further research.

This study found an overall rate of instrumental deliveries of 10.7%. Clinician members of the Choices and Challenges in Changing Childbirth Research Network report that training in obstetrics in the country is de-emphasising instrumental delivery, and the overall medical environment encourages caesarean section over instrumental deliveries. There were also significant differences in use of instrumental deliveries between public and private hospitals, and between those in Beirut/its suburbs and the rest of the country. Although no significant difference was noted between hospitals that were or were not accredited in the overall rate of instrumental deliveries, significantly higher use of forceps was found in accredited hospitals and significantly lower use of vacuum was found in accredited hospitals compared with non-accredited hospitals. The findings concerning human resources indicate that, at least in terms of overall numbers of providers to annual numbers of deliveries at the hospital level, midwives with childbirth privileges appear to have a higher load than obstetricians despite their lower professional status, which is likely explained by the fact that the obstetricians’ role is more focused on high-risk cases. However, the authors do not have data on the degree of assistance provided by other categories of health workers on the maternity ward, which will be further explored in the qualitative component of the research. The fact that there was no significant difference in this ratio between the public and private sectors and between Beirut/its suburbs and outside the capital indicates that this pattern appears to hold across the country.

In terms of the availability of essential equipment for safe maternal and newborn health care, the data indicate that both infusion pumps and fetal monitoring equipment are readily available across the public and private sectors, and in both Beirut and the rest of the country. The findings need to be considered in light of the above-noted high level of labour augmentation reported in Lebanon by clinicians within the Network – an issue that will be further explored in a planned survey of heads of maternity wards. This raises questions of safety for the one hospital lacking fetal monitors and the 17.5% of reporting hospitals that lacked infusion pumps about whether the potential effects on mother and newborn of labour augmentation are being monitored safely.

Moreover, there are large variations in the availability of NICUs (although the differences between hospitals in Beirut/suburbs and the rest of the country were found to be non-significant). Over half of hospitals responding to that question did not have an NICU. The fact that no significant variations between hospitals in Beirut/suburbs and the rest of the country in the provision of equipment and human resources, however, may be because less endowed hospitals were less likely to respond; indeed, the response rate of hospitals in less privileged areas of the country was lower (data by region not shown). This issue needs further research because of the noted predominance of the private sector in Beirut and its suburbs.

The finding that nearly half of responding hospitals lack an NICU needs to be further studied in context. Unlike in many health systems, the provision of NICUs is not regulated by the Lebanese Government to ensure a rational provision according to geography or population concentration (Khalid Yunis, Neonatologist, personal communication, 23/03/2010). NICUs are a major investment for hospitals to make, and there is no need for every hospital to have one if an adequate referral system exists. Nevertheless, anecdotal information from an obstetrician-gynaecologist in our network indicates that when a hospital lacks a NICU, it becomes complicated to transfer both mother and infant to a hospital supplied with one. This is mainly because the acceptance of the patient dyad is contingent upon the patient's mode of payment. In the Lebanese health system, this responsibility is not systematised but rather depends on a case-by-case negotiation by clinicians (Reem Abu Rustum, personal communication, 14/09/2009). The processes and problems in referral between hospitals for maternal/neonatal health have not been researched in Lebanon, and indeed internationally, this has been identified as an area in need of further research (Murray and Pearson, 2006).

## Limitations

These findings need to be qualified by the fact that under half of eligible hospitals responded to the survey and that limited information is available on the non-respondents; moreover, the response rate for the public sector was higher than that for the private sector, and was lower for less privileged areas of the country. There is a potential bias in the results, as noted, in that hospitals with better quality or higher levels of provision may have been more likely to respond. However, this bias is unlikely to affect the reported high caesarean section rate as capacity to perform caesarean section is high and widespread in Lebanese hospitals irrespective of size and location. Nevertheless, the nearly half response rate allows the authors to make some conclusions about hospitals in Lebanon with these provisos. Secondly, as the questionnaire was self-administered, some respondents may have misunderstood questions and there is potential for misreporting, although as noted, all respondents were knowledgeable about the maternal health services in the reporting hospitals. Data collected through the survey will be verified in the process of conducting structured interviews with heads of maternity wards in subsequent stages of the study described above. Thirdly, analysis by accreditation status was presented, but the last round of accreditation was in 2004 and therefore there is the possibility that hospital provision has changed since that date. Finally, a limitation in the scope of this study is its focus on provision of care and hospital-based statistics, not on the perspective of women who deliver in the maternity wards.

## Implications for practice and further research

This study confirms the importance of looking at the health system context of maternal health including mode of payment, distribution of services by sector and geographic region, and human resources. The lack of regulation over the expansion and distribution of maternal health services in Lebanon and the market incentives to invest in technologically sophisticated care, at the potential expense of training and oversight of human resources, is likely to be a factor contributing to the escalation of care illustrated by the very high reported hospital caesarean section rates. Moreover, understanding the predominant mode of payment for deliveries in different settings illuminates financial incentives to hospitals of quality improvement, and the scope for the MOPH, as the most significant single source of funding for childbirth overall, to influence the quality of care. A specific mapping of hospital-level provision and health outcome data allows us to ground recommendations for improvement in the context of what is actually provided.

These findings point to the importance of maternal health specialists engaging with health policy makers in the country and in other settings on the question of how health systems can support both the adoption of evidence-based interventions and, ultimately, better maternal and perinatal health outcomes. Given that there are other areas of maternal health care where lack of evidence-based care has been identified in Lebanon, in addition to caesarean section (Khayat and Campbell, 2000), there is an opportunity to inform the accreditation process with a broader range of appropriate indicators of quality of care in maternal health care at the hospital level. The lack of hospital-based metrics of quality of care has been noted as an urgent research gap internationally (Say et al., 2009), and is important to policy makers given that maternity care accounts for a high proportion of hospital services.

## Figures and Tables

**Table 1:** Latest nationally representative indicators on maternal health in Lebanon – PAPFAM 2004 (for all births in five years preceding survey).

Indicator	%
Childbirth by skilled attendant	98.2
Childbirth by unskilled attendant	1.8
Place of childbirth	
Private hospital or clinic	80.1
Public hospital	11.9
Private doctor	2.8
Home	2.4
Non-governmental health centre	1.2
General health centre	0.7
Maternal mortality ratio (per 100,000 live births)	88
Made five or more antenatal care visits	70.5
Antenatal care by medical doctor	93.6
Antenatal care by nurse/midwife	2.2
Proportion of births by caesarean section	23.2
Had at least one postnatal check up	51.6

*Source*: PAPFAM survey (Tutelian et al., 2007).

**Table 2:** Numbers of deliveries, live births and stillbirths by type and region of hospitals which responded to the survey.

Type of hospital	Hospital region	Number of deliveries (%)	Number of live births (%)	Number of stillbirths (%)
Public	Beirut and close suburbs	1464 (4.1)	1428 (4.0)	33 (6.8)
	Other regions	6504 (18.1)	6054 (17.1)	87 (17.9)
Private	Beirut and close suburbs	11,849 (33.0)	11,717 (33.2)	144 (29.6)
	Other regions	16,066 (44.8)	16,132 (45.7)	222 (45.7)

Total		35,883	35,331	486

**Table 3:** Job titles of survey respondents.

Survey respondents within study hospitals	n
Hospital/medical director	4
Head/in charge of obstetrics-gynaecology department	9
Head of childbirth unit	5
Hospital administrators (executive officer/medical doctor assistant/administrative assistant, financial manager, quality improvement coordinator/human resources manager/medical records officer/information technology officer/ER director)	11
Midwife or midwife in charge	10
Nurse or nurse in charge	12
Head of paediatric/maternal and baby health departments	2

**Table 4:** Percentage of deliveries by type of hospital.

Type of hospital	Number of deliveries	Percentage of all deliveries (n=35,883) (%)
Public	7968	22.2
Private	27,915	77.8

Total	35,883	100

**Table 5:** Distribution of the different modes of payment by type and region of hospital.

	MOPH	NSSF	Self-payers	Private insurance	Army	Other funds	Internal security	COOP	Total modes of payment
Type of hospital									
Public	85.3%	5.1%	5.2%	0.1%	2.2%	1.2%	0.7%	0.3%	8008
Private	21.9%	34.8%	13.2%	10.6%	8.1%	6.9%	2.5%	2.0%	26,060

Hospital region									
Beirut and close suburbs	18.8%	38.0%	15.8%	15.0%	2.2%	7.4%	1.5%	1.3%	11,523
Outside Beirut	46.1%	22.6%	9.0%	4.6%	9.0%	4.6%	2.4%	1.8%	22,545
Total	36.8%	27.8%	11.3%	8.1%	6.7%	5.5%	2.1%	1.6%	34,068

MOPH, Ministry of Public Health; NSSF, National Social Security Fund; CSC, Civil Servants Cooperative. Other military schemes include the Internal Security Forces, General Security Forces and State Security Forces. Note: The total modes of payment exceed the number of deliveries as delivery expenses could be covered by more than one source of funding.

**Table 6:** Distribution of caesarean section rates by hospital type and region.

	Mean caesarean section rate (SD)	*p*-Value
Hospital type		
Public	40.2 (14.0)	0.872
Private	41.0 (15.0)	

Hospital region		
Beirut and close suburbs	38.7 (11.4)	0.488
Other regions	41.7 (16.0)	

Sample hospital caesarean section prevalence	40.8 (14.7)	

**Table 7:** Distribution of vaginal instrumental deliveries by type of hospital and by hospital location.

	Mean (SD)	*p*-Value
Percentage of vaginal instrumental deliveries		
Type of hospital		
Public	3.9 (5.1)	0.002
Private	12.4 (12.5)	

Hospital region		
Beirut and close suburbs	18.0 (13.5)	0.001
Other regions	6.9 (9.1)	

Total (n=50)	10.7 (11.9)	

**Table 8:** Distribution of indicators of childbirth practices and availability of equipment according to accredited versus non-accredited status of private hospitals.

	Hospitals that passed (n=35)	Hospitals that failed (n=6)	*p-*Value^a^
Percent caesarean section rate (mean, SD)	44.4 (13.7)	31.4 (7.9)	0.031
Percent use of instrumental deliveries (mean, SD)	13.1 (13.0)	7.0 (7.1)	0.275
Percent use of instrumental deliveries by forceps (mean, SD)	41.0 (36.4)	8.3 (12.5)	0.004
Percent use of instrumental deliveries by vacuum (mean, SD)	56.7 (36.5)	91.7 (12.5)	0.002
Number of fetal monitors per childbirth per day (mean, SD)	3.0 (3.2)	9.2 (13.9)	0.324
Number of IVAC per childbirth per day (mean, SD)	2.7 (4.4)	2.3 (2.0)	0.825
Number of NICUs per childbirth per day (mean, SD)	0.3 (0.3)	0.9 (2.1)	0.540
Number of deliveries per labour bed per day (mean, SD)	0.7 (0.5)	0.2 (0.2)	0.000
Number of deliveries per childbirth room per day (mean, SD)	1.1 (0.9)	0.4 (0.4)	0.079

NICU, neonatal intensive care unit.

a On the basis of independent samples *t*-test.

**Table 9:** Average number of deliveries annually by number of health providers in hospitals, 2008.

Hospital type	Hospital region	Obstetrician/gynaecologist (mean, SE)	Midwife with childbirth privileges (mean, SE)

Public	Beirut and close suburbs	38.5	133.1
	Other regions	43.6 (8.1)	127.4 (32.6)
Private	Beirut and close suburbs	32.4 (4.3)	112.7 (21.2)
	Other regions	51.7 (12.2)	190.0 (72.9)

**Table 10:** Distribution of the availability of fetal monitors, infusion pumps, neonatal intensive care units (NICUs) labour beds and childbirth rooms by type of hospital and by hospital location.

	Mean number of fetal monitors per childbirth (SD)	Mean number of IVAC per childbirth (SD)	Mean number of NICUs per childbirth (SD)	Mean number of deliveries per labour bed (SD)	Mean number of deliveries per childbirth room (SD)
Hospital type					
n	55	56	46	53	55
Public	3.1 (2.4)	2.3 (2.3)	0.1 (0.2)	0.6 (0.9)	0.8 (0.9)
Private	4.0 (6.1)	2.7 (4.2)	0.4 (0.9)	0.6 (0.5)	1.0 (0.9)
*p*-Value	0.627	0.712	0.303	0.927	0.660

Hospital region					
Beirut and close suburbs	3.4 (2.5)	2.6 (2.0)	0.6 (1.3)	0.6 (0.5)	1.0 (0.7)
Other regions	4.0 (6.4)	2.7 (4.4)	0.2 (0.3)	0.6 (0.6)	0.9 (0.9)
*p*-Value	0.680	0.925	0.160	0.866	0.712

Total	3.8 (5.5)	2.7 (3.8)	0.3 (0.8)	0.6 (0.6)	0.9 (0.9)

## Footnotes

1 See http://fhs.aub.edu.lb/cccc/for a description of the Network and list of publications.

2 For findings from Egypt on almost universal use of oxytocin, the majority of which is not indicated, see Khalil et al. (2004).

3 One exceptional area where data are available is a database on neonatal health which was the initiative of one neonatologist in the private sector and now includes a network of over 20 participating hospitals (see http://www.ncpnn.org/users/index.asp). See, for example, Tamim et al. (2007).

4 One medical centre was excluded as it reported that it was exclusively accepting planned caesarean section deliveries during renovation of the maternity ward at the time of the survey.

5 One hospital was excluded as it only conducts caesarean sections and thus the total included was 57.

6 In Egypt, a country of over 80 million, recent population-based data indicate that 27.6% of preceding births were delivered by caesarean section (El-Zanaty and Way, 2009).
